# Immune functions of avian erythrocytes: a comprehensive review from basic biology to pathogen-induced responses

**DOI:** 10.3389/fimmu.2025.1675279

**Published:** 2025-11-20

**Authors:** Fujuan Cai, Yuzhen Wang

**Affiliations:** 1 School of Life Sciences, Inner Mongolia Key Laboratory of Biomanufacturing Technology, Inner Mongolia Agricultural University, Hohhot, China; 2 School of Statistics and Mathematics, Inner Mongolia University of Finance and Economics, Hohhot, China

**Keywords:** avian erythrocytes, immune responses, toll-like receptors (TLRs), cytokines, oxidative stress, phagocytosis

## Abstract

Avian erythrocytes, traditionally perceived as mere oxygen transporters, are increasingly acknowledged as integral components of the immune system. Unlike enucleated erythrocytes of mammals, avian erythrocytes retain functional nuclei and organelles, allowing them to produce immune molecules like cytokines and antimicrobial peptides essential for pathogen recognition and clearance. This review provides a comprehensive elucidation of the molecular mechanisms underpinning the immune functions of avian erythrocytes and their responses to infections induced by viruses, bacteria, fungi, *mycoplasma*, and parasites. Through a systematic analysis of literature spanning the past 16 years, we synthesize evidence regarding the involvement of avian erythrocytes in Toll-like receptor (TLR) signaling, oxidative stress responses, phagocytosis, immune adhesion, and apoptosis-mediated immune evasion. We further demonstrate their pivotal role in anti-infective immunity, underscoring their ability to synthesize and secrete immune molecules, effectively capture and eliminate pathogens, and precisely regulate inflammatory responses and immune homeostasis. Finally, we identify existing knowledge gaps and underscore the necessity for further research to fully elucidate erythrocyte-mediated immune mechanisms and their potential applications in the prevention and control of avian diseases.

## 1 Introduction

As the predominant cell type within the circulatory system, erythrocytes are increasingly acknowledged for their roles in host immunity. Current research suggests that erythrocytes contribute to immune responses through cytokine production, binding of free mitochondria, and modulation of the canonical Nuclear Factor kappa-light-chain-enhancer of activated B cells (NF-κB) signaling pathway (1–3). Unlike their mammalian counterparts, avian erythrocytes retain their nucleus and organelles, which endow them with distinct immunological functions. Mammalian erythrocytes primarily mediate immune functions by binding inflammatory molecules and displaying phagocytic-like activity. In addition to these mechanisms, avian erythrocytes possess the ability to perform transcription and translation, allowing them to respond to pathogen-associated molecular patterns (PAMPs) and participate in immune reactions via pattern recognition receptors (PRRs) (4). Studies have shown that chicken erythrocytes can engage in immune responses by upregulating specific cytokines upon viral infection (5), while goose erythrocytes demonstrate increased cytokine expression following bacterial stimulation (6). Moreover, genes associated with the complement system on the surface of chicken erythrocytes are swiftly activated upon adherence to *Escherichia coli* (*E. coli*) (7), while goose erythrocytes exhibit phagocytic or adherent activity towards bacteria (6). Although the immune functions of human erythrocytes are well-documented in the literature, the immune functions of chicken erythrocytes have not been comprehensively explored. This review seeks to provide a comprehensive overview of the immune mechanisms of avian erythrocytes and their immunoresponsive behavior in response to pathogen infection. It elucidates the innate immune functions of avian erythrocytes by synthesizing current knowledge on their molecular mechanisms.

## 2 Methods

This review utilized a systematic literature search approach to explore the immune functions of avian erythrocytes, encompassing both fundamental biological aspects and responses to pathogenic challenges. Relevant studies were identified through comprehensive searches in scientific databases such as Scopus, Web of Science, ScienceDirect, PubMed, CABI, and Google Scholar. The literature search employed various combinations of keywords, including “erythrocyte,”“avian,”“immunity,”“TLR,”“oxidative stress,”“phagocytosis,”“apoptosis,”“cytokine,” and “C3b.” This review provides a comprehensive examination of a diverse array of avian species, encompassing prominent domestic fowl such as the chicken (*Gallus gallus domesticus*), goose (*Anser cygnoides/Anser anser*), turkey (*Meleagris gallopavo*), and Japanese quail (*Coturnix japonica*); common waterfowl including the mallard (*Anas platyrhynchos*) and spot-billed duck (*Anas zonorhyncha*); as well as significant wild and model avian species such as the zebra finch (*Taeniopygia guttata*), red-headed bunting (*Emberiza bruniceps*), coal tit (*Periparus ater*), great tit (*Parus major*), white stork (*Ciconia ciconia*), golden pheasant (*Chrysolophus pictus*), fieldfare (*Turdus pilaris*), common blackbird (*Turdus merula*), European turtle dove (*Streptopelia turtur*), and rock dove (*Columba livia*). The review prioritizes peer-reviewed journal articles, with a particular emphasis on literature pertaining to immune-related research on avian erythrocytes.

The initial search process identified 2,200 records. After the removal of duplicates, 1,244 unique publications were retained. An abstract-level assessment led to the exclusion of 852 studies that either lacked quantitative data or did not clearly define exposure parameters, leaving 392 articles for the eligibility evaluation stage. Full texts of 300 studies were subsequently reviewed, with stringent inclusion criteria applied: studies were required to explore the immune functions of avian erythrocytes (such as receptor expression, cytokine production, and complement activity) and to be original research or systematic reviews published in English. Exclusion criteria encompassed studies focusing exclusively on the traditional physiological functions of erythrocytes and non-peer-reviewed literature. Following the exclusion of reports with incomplete methodologies, 93 studies were ultimately selected for comprehensive analysis. This review predominantly focuses on literature published between 2010 and 2025.

## 3 Structure and molecular of avian erythrocytes

The retention of the nucleus in avian erythrocytes imparts a unique structural and functional complexity to these cells. These distinct cellular attributes, coupled with their expression of immune recognition and effector molecules (see **Table 1**), form the structural foundation for their pivotal roles in immune function.

**Table 1:** The immune components of avain erythrocytes, and its relevance to infection state.

Erythrocyte components	Associated molecule	Immune functions	Relevant infection states
TLR	TLR3-polyI:C	Transduce viral signals	MDV (8)
	TLR4-LPS	Transduce bacteria signals	*S. Aureus, A.hydrophila, E. coli* (6)
CR1	C3b	Immune adhesion	IBDV (9)
MHC II	exogenous antigens	Presenting antigen to CD4^+^ T cells	MDV (8)
HDPs	AvBDs	Kill pathogenic micro-organisms	MDV (10)
	Histone		vancomycin-resistant *enterococci* (11)
IL	IL-1β, IL-6, IL-8	Transduce immune signals	*S. Aureus, A.hydrophila, E. coli* (6)
	IL-7, IL-12, IL-13		MDV (8)
IFN	IFN-α, IFN-β	Interferon Signaling Cascade	MDV (8)
	IFN-γ		*Candida albicans* (12)

TLR, Toll-like Receptor; CR1, Complement Receptor type 1; MHC II, Major Histocompatibility Complex Class II; HDPs, Host defense peptides; IL, interleukin; IFN, interferon; polyI:C, Polyinosinic-polycytidylic acid; LPS, Lipopolysaccharide; C3b, Complement Component 3b; AvBDs, avian β-defensins; MDV, Marek's disease virus; IBDV, Infectious bursal disease virus; *S. Aureus*, *Staphylococcus aureus*; *A.hydrophila*, *Aeromonas hydrophila*; *E. coli*, *Escherichia coli.*

### 3.1 Structure of avain erythrocytes

#### 3.1.1 Cell nucleus

Mature avian erythrocytes possess an intermediate diameter, larger than that of mammalian erythrocytes but smaller than that of amphibian erythrocytes (13). Morphologically, avian erythrocytes, such as those found in chickens, are elliptical or flattened ovoids, measuring approximately 12 μm in diameter (14), in contrast to the typical biconcave disc shape of mammalian erythrocytes. The presence of the nucleus not only facilitates the transcription and translation of proteins essential for gas exchange but also supports the expression of proteins involved in immune responses.

#### 3.1.2 Mitochondria

Avian erythrocytes retain nuclei and contain organelles within their cytoplasm (15). Research conducted by Antoine Stier et al. (16) has demonstrated that mitochondria persist in the cytoplasm of zebra finch (*Taeniopygia guttata*) erythrocytes even after the removal of hemoglobin. Their study further revealed that these mitochondria are capable of respiration, similar to mammalian erythrocytes, thereby challenging the previously held view that avian erythrocyte energy metabolism relies exclusively on the pentose phosphate pathway and glycolysis. This active mitochondrial respiration provides a structural basis for their involvement in various biological processes. Notably, during migration, red-headed buntings (*Emberiza bruniceps*) exhibit significant increases in hematocrit, erythrocyte surface area, and mitochondrial membrane potential (MMP). Simultaneously, there is a reduction in reactive oxygen species (ROS) levels and the proportion of apoptotic erythrocytes. The expression of antioxidant genes, such as Superoxide Dismutase 1 (*SOD1*) and nitric oxide synthase 2 (*NOS2*), the cluster of differentiation 36 (CD36), and key metabolic genes is significantly upregulated (17). These observations suggest adaptive modifications in mitochondrial function and erythrocyte apoptosis in response to the energetic demands associated with migration. To endure the cold temperatures of winter, coal tits (*Periparus ater*) and great tits (*Parus major*) exhibit enhanced erythrocyte mitochondrial respiratory rates and increased mitochondrial volume. Overall, these findings imply that birds adjust mitochondrial metabolic activity within erythrocytes as an adaptive mechanism to cope with environmental stressors.

#### 3.1.3 Hemoglobin

Upon release from erythrocytes following hemolysis, hemoglobin exhibits peroxidase-like activity, generating ROS that contribute to its antibacterial function. This antimicrobial activity is not influenced by the blood donor’s blood group, age, or sex. The antibacterial properties primarily originate from the protein moiety of hemoglobin, as activity persists even after the removal of the heme prosthetic group (18). Additionally, a cationic alpha-helix formed by the thirty carboxyl-terminal amino acids of the hemoglobin beta subunit demonstrates efficacy against pathogens including *E. coli*, *Staphylococcus aureus* (*S. aureus*), Candida albicans (*C. albicans*), and *Bacillus subtilis* (*B. subtilis*) (18, 19). Hemoglobin requires the oxygenated environment of blood to exert its antimicrobial effects, and the dynamic nature of blood circulation positions erythrocytes as the primary mediators of this antibacterial activity within the bloodstream.

#### 3.1.4 Histones

Rose-Martel and Hincke (20) have identified histones as cationic antimicrobial peptides (CAMPs). Through electrophoretic mobility shift assays, they demonstrated that CAMPs exhibit antibacterial activity by targeting conserved negatively charged components within pathogen membranes. Their study further elucidated that antimicrobial histones derived from chicken erythrocytes specifically bind to bacterial lipopolysaccharide (LPS) and lipoteichoic acid (LTA). Additionally, their findings indicate that a purified mixture of histones (H1, H2B, H2A, H3, H4, and H5) isolated from chicken erythrocytes possesses significant growth-inhibitory effects against *B. subtilis*, *S. aureus*, *Salmonella enterica* serovar Typhimurium, *Pseudomonas aeruginosa* (*P. aeruginosa*), and *E. coli*. These findings demonstrate that histones exhibit significant antibacterial properties against both Gram-negative and Gram-positive bacteria. In a complementary structural investigation, Davie et al. (21) utilized an advanced native fractionation technique to analyze histone modifications, histone variants, atypical nucleosomes (U-shaped nucleosomes), and other chromatin structural characteristics, such as open chromatin. Their research included the pioneering mapping of histone H4 asymmetrically dimethylated at arginine 3 (H4R3me2a) and histone H3 symmetrically dimethylated at arginine 2 (H3R2me2s), which are the products of protein arginine methyltransferases (PRMT) 1 and 5, respectively.

### 3.2 Immune recognition molecules

#### 3.2.1 Toll-like receptors

Avian erythrocytes inherently express a wide range of TLRs on their surface. Studies have shown that chicken erythrocytes consistently express transcripts encoding *TLRs 1*, *2*, *3*, *4*, *5*, *7*, *15*, and *21* (4, 22). TLR3 is known to recognize viral double-stranded RNA (dsRNA) and triggers innate immune responses through a myeloid differentiation primary response 88 (MyD88)-independent pathway, primarily mediated by the adaptor protein TRIF. Furthermore, TLR3 can be activated by polyinosinic:polycytidylic acid (poly(I:C)), a synthetic analog of dsRNA. Activation of TLR3 specifically induces the production of interferon-β (IFN-β) (4, 23). TLR4, on the other hand, is specifically activated by LPS, a key component of Gram-negative bacterial membranes. This receptor predominantly transmits signals via the MyD88-dependent pathway, serving a crucial function in the avian immune response to bacterial infections. Activation of TLR4 leads to the induction of pro-inflammatory cytokines, including tumor necrosis factor (TNF-α), interleukin-1 (IL-1β), IL-6, and IL-12, thereby facilitating antimicrobial defense (24). TLR21, a distinctive Toll-like receptor in chickens, plays a significant role in the immune recognition of microbial infections. In contrast to most TLRs, TLR21 specifically identifies unmethylated CpG oligodeoxynucleotide (CpG ODN) motifs, which are characteristic of bacterial and viral genomes. TLR21 also signals through MyD88 to elicit TNF-α-mediated antimicrobial responses (25). Research on erythrocytes of the common carp (*Cyprinus carpio*) indicates an upregulation of TLR4 and TLR9 in response to *Aeromonas hydrophila* (*A. hydrophlia*) infection, accompanied by the secretion of TNF-α and IFN-γ (26). Currently, there are no studies documenting TLR9 expression in chicken erythrocytes.

#### 3.2.2 Complement receptors

The currently identified complement receptoron the erythrocyte membrane include complement receptor 1 (CR1), also known as the C3b receptor, and CR3 (27). Studies have demonstrated that CR1 receptors are expressed on the surfaces of both human reticulocytes and normal erythrocytes (28). Furthermore, research has identified the presence of C3b receptors (C3bR) on erythrocytes from various avian species, such as the golden pheasant (*Chrysolophus pictus*), ostrich (*Struthio camelus*), goose (*Anser* spp.), quail (*Coturnix coturnix*), gray junglefowl (*Gallus sonneratii*), Yunnan mallard duck (*Anas platyrhynchos*), and Cherry Valley duck (*Anas platyrhynchos domesticus*) (29). The CR1 receptors on erythrocytes are not aggregated but are instead dispersed across the membrane. This dispersed distribution facilitates the binding of complement-tagged particles. While the dispersed arrangement of CR1 is likely advantageous for capturing immune complexes (ICs), the receptor clustering that occurs upon binding may inhibit erythrophagocytosis during the transfer of ICs to macrophages by maintaining localized phagocytic stimuli (30). Research has shown that the immunological functions of erythrocytes are primarily mediated through the CR1 on their membrane, which directly influences the clearance of circulating ICs (31).

#### 3.2.3 MHC

Research indicates that chicken erythrocytes express complex classI (*MHC I*), with cells harboring a greater number of *MHC* gene copies displaying increased expression levels (32). Additionally, studies have shown that chicken erythrocytes also express *MHC II*. Importantly, during infection with various pathogens, the expression of *MHC II* is subject to modulation (8, 22, 33). Furthermore, investigations have identified conserved MHC I and II epitopes on the surface of both human erythroid precursors and erythrocytes. MHC class II epitopes derived from antigens associated with the pre-erythrocytic, erythrocytic, or sexual stages of *Plasmodium falciparum* provide coverage ranging from 98.5% to 100% against the parasite and include all variants of malaria pathogens that evade the immune system (34).

### 3.3 Immune effector molecules

#### 3.3.1 Cytokines

Avian erythrocytes demonstrate precise PAMP and PRR responses at the transcriptomic level and are capable of producing specific signaling molecules, such as cytokines, to initiate immune responses. Research indicates that exposure of chicken erythrocytes to polyinosinic:polycytidylic acid (poly I:C) and ODN leads to an upregulation in the expression of type I IFNs, specifically IFN-alpha (IFN-α) and *IFN-β* (4). These interferons play a crucial role in establishing an antiviral state by inhibiting viral transcription and translation, promoting apoptosis in infected cells, and activating antigen-presenting cells. Additionally, the chemokine CCL4 was observed (13). Transcripts of the interferon-induced gene 2’-5’ oligoadenylate synthetase were also detected, which initiates the RNase L pathway to degrade viral RNA (35). Moreover, high-dose poly I:C treatment significantly induced the expression of *IL-8* transcripts (36). Furthermore, the expression of cytokines such as *IL-6*, *IL-1β*, *TNF-α*, *IL-7*, *IFN-β*, *IL-12*, *IL-13*, and *IFN-α* was detected in chicken erythrocytes (3, 8). Similarly, the expression of *IL-6*, *IL-1β*, and *IL-8* was identified in goose erythrocytes (6).

#### 3.3.2 Superoxide dismutase

Research has shown that co-culturing goose erythrocytes with bacteria such as *S. aureus*, *Streptococcus aqua*, and *E. coli* significantly upregulates the expression of SOD, suggesting that this response fulfills an antioxidant role (6). Furthermore, studies have demonstrated that human erythrocytes contain SOD, with enzyme levels significantly elevated in patients with thyroid nodules compared to healthy controls (37). Similarly, research has indicated that mouse erythrocytes inherently possess SOD, which exhibits a notable decrease following LPS stimulation (38).

The maintenance of sustained SOD activity is crucial for preserving intracellular redox homeostasis, thereby preventing the excessive accumulation of ROS that could potentially disrupt immune signaling pathways, such as the TLR and interferon pathways. This preservation is essential for ensuring effective antiviral and antibacterial responses (39). In contrast to mammalian erythrocytes, avian erythrocytes retain functional mitochondria and nuclei, which facilitate the continuous synthesis of antioxidant enzymes. This capability allows avian erythrocytes to maintain immunomodulatory functions during episodes of infection or oxidative stress. Consequently, the stability of SOD may serve as a potential biomarker for assessing disease resistance in avian species and provides a theoretical basis for developing strategies in disease-resistant breeding and antioxidant-based therapeutic interventions.

#### 3.3.3 Host defense peptides

Yacoub et al. (40) demonstrated that the synthetic chicken host defense peptides, β-defensin peptide-4 (sAvBD-4) and sAvBD-10, exhibit significant antimicrobial activity against a broad spectrum of bacterial and fungal pathogens. In a related study, Niu et al. (10) identified the constitutive expression of transcripts for eight avian β-defensins (*AvBDs*) (*AvBD1*-*AvBD7* and *AvBD9*) as well as liver-expressed antimicrobial peptide-2 (*LEAP-2*) in normal chicken erythrocytes. Upon infection with Marek’s disease virus (MDV), chicken erythrocytes appear to counteract the viral infection through the upregulated expression of *AvBD2*, *AvBD4*, and *AvBD7*, while the expression levels of *AvBD1*, *AvBD6*, and *AvBD9* were significantly diminished. These findings suggest that the *AvBDs* expressed in chicken erythrocytes play a crucial role in the host immune response elicited by MDV infection.

## 4 Immune functions of avian erythrocytes

**Figure 1:**
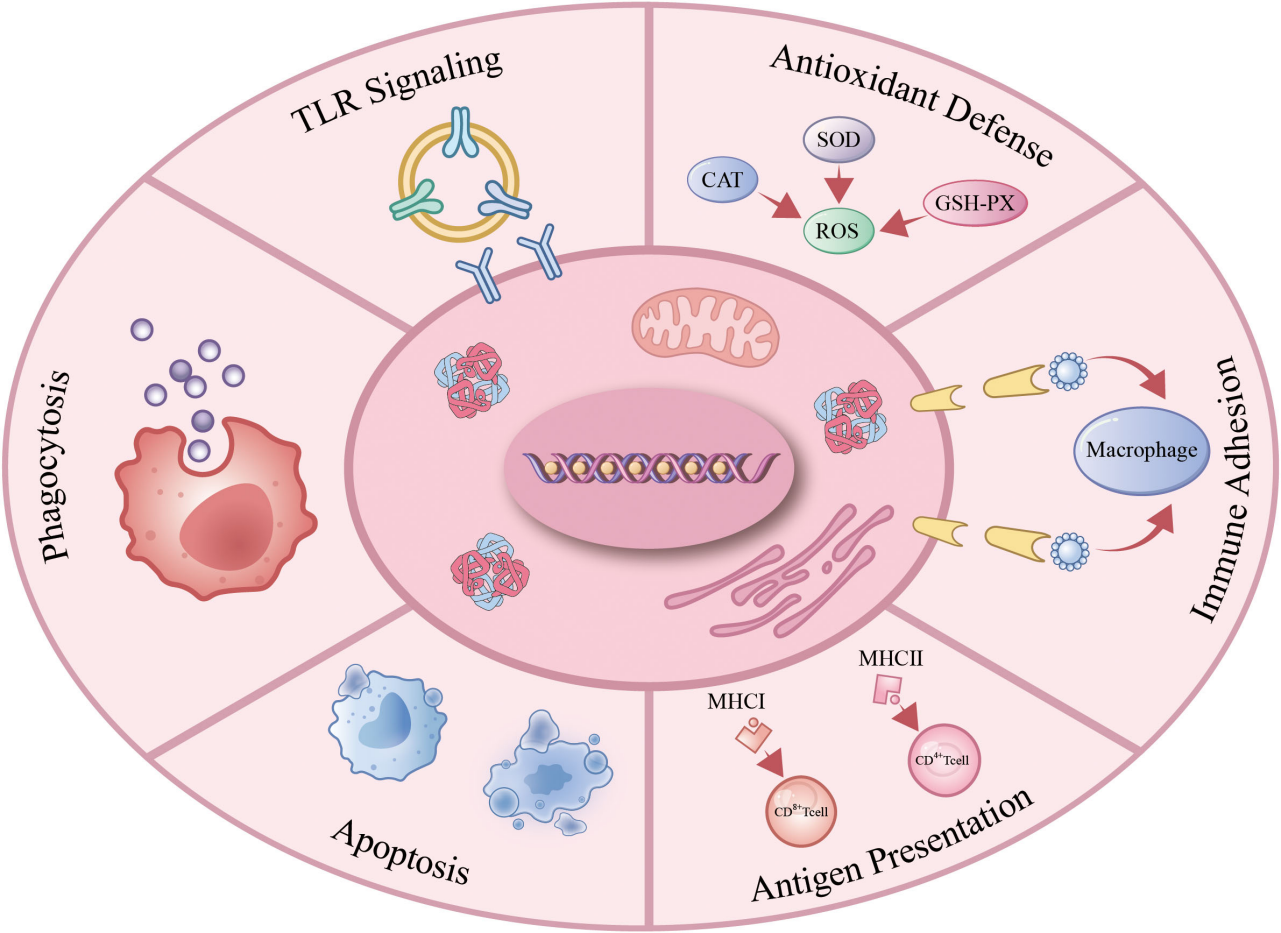
Immune function of avain erythrocytes. Avian erythrocytes operate as immunocompetent cells by: activating TLR-dependent pathogen sensing; scavenging ROS under oxidative stress; mediating CR1/CR3-facilitated immune adhesion; presenting antigens via constitutive MHC expression; executing immunomodulatory apoptosis; and directly phagocytizing pathogens or enhancing opsonophagocytosis. .

As understanding of the immunological functions of avian erythrocytes deepens, research reveals their involvement in immune defense through multiple processes. These include activation of TLR signaling pathways, responses to oxidative and heat stress, execution of phagocytic functions, participation in immune adhesion, and evasion of immune surveillance via apoptosis. Collectively, these mechanisms enable avian erythrocytes to counteract pathogen invasion and maintain immune homeostasis (**Figure 1**).

### 4.1 Activation of TLR signaling pathways

Studies have shown that stimulation of chicken erythrocytes with the TLR3 ligand poly(I:C) and the TLR21 ligand CpG ODN leads to an upregulation of type I interferon transcripts. However, only poly(I:C) treatment results in elevated *IL-8* transcript levels and increased nitric oxide production (4). Subsequent research by Morera et al. (41) identified distinct regulatory patterns of TLR expression in chicken erythrocytes: *TLR3* expression is significantly upregulated following stimulation with recombinant TNF (rTNF) but is downregulated after treatment with poly(I:C) and peptidoglycan (PGN). In contrast, *TLR21* expression is markedly increased upon stimulation with bacterial LPS and PGN, while it is reduced following exposure to poly(I:C) and rTNF. Furthermore, research indicates that chicken TLR21 (chTLR21) activates NF-κB and induces a robust type I interferon response in chicken macrophages, akin to the signaling cascade observed with mammalian TLR9 activation. Notably, chTLR21-mediated transcription of IFN-β is dependent on both NF-κB and IRF7 signaling pathways, yet it is independent of TBK1 kinase, a distinctive feature that contrasts with mammalian TLR9 signaling (42).

### 4.2 Antioxidant defense

Under conditions of oxidative stress, avian erythrocytes demonstrate a substantial antioxidant response. Pineda-Pampliega et al. (43) reported that administering the antioxidants tocopherol (vitamin E) and selenium to White Stork (*Ciconia ciconia*) nestlings significantly reduced the average rate of erythrocyte telomere length shortening. This study indicates that erythrocytes in White Stork nestlings actively engage in antioxidant stress responses. The erythrocyte membrane, being rich in polyunsaturated fatty acids, coupled with the role of free radicals in oxygen transport, renders erythrocytes highly susceptible to free radical exposure. Zhang et al. (44) demonstrated that exposure of chicken erythrocytes to the free radical generator 2,2’-azobis (2-methylpropionamidine) dihydrochloride (AAPH) resulted in severe hemolysis, decreased SOD activity, and increased malondialdehyde (MDA) levels. Furthermore, Rani A et al. (45) observed that the herbicide Ethoxysulfuron induced nuclear abnormalities in the erythrocytes of male Japanese quail, a phenomenon potentially linked to factors such as mitochondrial oxidative stress. In contrast, the antioxidants curcumin and bisdemethoxycurcumin were observed to decrease hemolysis in erythrocytes treated with AAPH, restore SOD activity, and inhibit lipid peroxidation (LPO). Furthermore, the assessment of hemolysis time in the presence of an oxidizing agent may serve as an indirect measure of the antioxidant capacity of erythrocytes (46).

The investigation conducted by Goodchild and DuRant (47) revealed that the exposure of zebra finch (*Taeniopygia guttata*) erythrocytes to excessive hydrogen peroxide led to a dose-dependent elevation in heme degradation products (HDPs), which are recognized as biomarkers of oxidative stress. Furthermore, the study demonstrated a positive correlation between the fluorescence intensity of HDPs and both compromised erythrocyte membrane integrity and heightened erythrocyte osmotic fragility. This research implies that ROS may indirectly impair erythrocyte membrane integrity through the production of membrane-associated hemoglobin degradation products. In parallel, Jin et al. (48) reported that endogenously generated hydrogen sulfide (H_2_S) facilitates oxidative phosphorylation and ATP biosynthesis in chicken erythrocytes. Collectively, these studies contribute to a more comprehensive understanding of the factors influencing erythrocyte health in avian species.

### 4.3 Apoptosis

Avian erythrocytes are equipped with apoptotic mechanisms that may facilitate immune evasion under specific physiological conditions. When exposed to external factors such as elevated temperatures, chemical toxins, oxidants, and viral pathogens, these erythrocytes undergo significant morphological changes and exhibit apoptotic features. In the case of pigeon erythrocytes exposed to hydrogen peroxide (H_2_O_2_), there is an increase in LPO, resulting in notable alterations in the phospholipid composition of the erythrocyte membrane (49). Chromatin dispersion analysis has demonstrated that high-temperature stress induces pronounced nuclear dissolution and fragmentation in pigeon erythrocytes, which are hallmarks of apoptosis (50). This membrane-nuclear dual-response mechanism allows avian erythrocytes to simultaneously detect the activation of apoptotic pathways and direct damage to genetic material (51). This dual perspective, encompassing both membrane morphology and nuclear events, offers an effective model for assessing the overall toxicity of exogenous substances in avian toxicology research. Szabelak et al. (52) found that when chicken erythrocytes were exposed to high temperatures, significant morphological changes occurred, accompanying activity of Caspase 3 and 7. The caspase pathway is a classical apoptotic signaling pathway.

Avian research offers potential strategies for safeguarding poultry against tibial dyschondroplasia (TD). Studies have indicated that during the initial stages of thiram-induced TD, erythrocytes show diminished expression of anti-apoptotic genes, including *Bcl-2, BAG-1, BAG-3*, and *STAT3*. This expression is restored during the recovery phase, suggesting a significant link between erythrocyte apoptosis and the progression of TD (53). Additionally, research has demonstrated that exposure to cadmium (Cd) results in abnormal erythrocyte morphology and nuclear anomalies, leading to dose- and time-dependent apoptosis, which is associated with oxidative stress and a reduction in MMP. It has been shown that Zn²^+^ and N-acetyl-L-cysteine (NAC) mitigate Cd-induced erythrocyte apoptosis through distinct pathways (54). Moreover, Zhang et al. (55) discovered in a chicken erythrocyte model that the free radical initiator AAPH induces an apoptosis-like phenotype, whereas natural antioxidants, such as curcumin, effectively inhibit this process by neutralizing free radicals and preserving membrane lipid homeostasis.

The mechanism by which avian erythrocytes achieve immune evasion through apoptosis has been extensively investigated in various studies. Studies suggest that the TLR signaling pathway, along with its associated genes, plays a crucial role in initiating cell apoptosis upon the detection of viral and toxic agents within the organism (56–59). Upon stimulation with poly(I:C) and CpG ODN, chicken erythrocytes exhibit increased expression of type I interferons, thereby facilitating the apoptosis of virus-infected cells (4). Empirical evidence indicates that the chicken anemia virus (CAV) induces anemia by infecting mature erythrocytes and triggering their apoptosis, with the severity of anemia being positively correlated with the viral load (60). In the thiram-induced tibial dyschondroplasia (TD) model in broilers, a significant upregulation in the expression levels of molecules including MDA5, MyD88, MHCII, TRAF6, TLR2, TLR3, TLR4, TLR5, and TLR7 was observed. This molecular alteration was associated with inhibited vascular invasion in the growth plate, resulting in energy metabolism disorders, chondrocyte apoptosis, and impaired endochondral ossification, thereby manifesting the characteristic pathological features of TD. The recombinant protein rGSTA3 has the potential to inhibit chondrocyte apoptosis by downregulating TLRs (33). Collectively, these studies indicate that apoptosis in avian erythrocytes, when subjected to external stressors, serves not only as a stress response but also as a mechanism for immune evasion. By undergoing apoptosis, erythrocytes can effectively mitigate excessive immune responses, thereby protecting themselves from immune clearance.

### 4.4 Phagocytosis

Avian erythrocytes exhibit phagocytic capabilities or promote phagocytosis. Research has demonstrated that chicken erythrocytes display significant phagocytic activity against 8.5-micrometer polystyrene beads, thereby enhancing pulmonary defense mechanisms (61). Confocal laser scanning microscopy has revealed that during migration, avian erythrocytes develop pseudopodia containing parallel arrays of actin filaments. Similar to leukocytes, alterations in the actin cytoskeleton of nucleated erythrocytes are critical for their migratory and phagocytic functions (62). Yang et al. (6) demonstrated that goose erythrocytes exhibit significant phagocytic or adhesive activity towards latex beads of a specific diameter. During the phagocytosis of these beads, notable vesicular structures were observed on the surface of the goose erythrocytes, with the cells forming invaginations to engulf the beads. This process was time-dependent and exhibited phagocytic efficiency comparable to that of macrophages and lymphocytes (63, 64). Additionally, goose erythrocytes displayed extensive phagocytic or adhesive activity against *Escherichia coli* and *S. aureus* within a defined time period (6). In chickens, erythrocytes facilitate the phagocytosis of *C. albicans* by monocytes/macrophages (Mo-MØ) (12). In human erythrocytes, phagocytic function may be associated with the expression of complement receptors on the cell surface. We propose that complement receptors may similarly play a role in the phagocytic activity of avian erythrocytes.

### 4.5 Heat stress responses

The study conducted by Szabelak A et al. demonstrated that short-term exposure of hen blood to temperatures ranging from 43 to 45 °C induces morphological alterations, enhances the activity of pro-apoptotic Caspase 3 and 7, and results in the presence of hemolyzed cells (52). An earlier investigation revealed that the combination of fisetin with probiotics ameliorates oxidative stress-related changes in broilers and mitigates erythrocyte fragility induced by heat stress (65). In research carried out by Greene et al. on the immune responses of various commercial chicken breeds—including those from conventional and high-yield production lines—under heat stress conditions, it was observed that, in contrast to the response of white blood cells, heat stress predominantly influenced the levels of TNF-α, C-C motif chemokine ligand 4 (CCL4), and C-C motif chemokine-like 4 (CCLL4) in erythrocytes. Furthermore, significant differences were identified in the expression of inflammasome genes (NLRP3, NLRC5, and NLRC3) among chickens from different production lines under heat stress conditions (66).

### 4.6 Immune adhesion

Avian erythrocytes demonstrate immune adhesion activity. Comparative research indicates that lymphocytes from chicks are more adept at forming spontaneous rosette formations with various avian erythrocytes than erythrocytes from other species, such as carp, bullfrogs, snakes, and certain mammals (67). Within the spectrum of avian erythrocytes, quail red blood cells (QRBCs) exhibit the highest propensity for rosette formation in the bone marrow and spleen (QRBC-rosette), whereas this occurrence is infrequently observed in the thymus and peripheral blood lymphocytes (PBLs) (67). It is hypothesized that the velocity of blood flow impedes the phagocytic function of leukocytes. As erythrocytes traverse the bloodstream, they acquire an electric charge due to the triboelectric effect, which facilitates the attraction and adhesion of bacteria to their surface. Subsequently, these bacteria are phagocytosed and neutralized by the oxygen present in hemoglobin. Erythrocytes play a crucial role in the clearance of pathogens from the bloodstream, while leukocytes are responsible for the phagocytosis and destruction of bacteria outside the circulatory system, specifically within tissues, lymph nodes, and areas of reduced lymphatic flow (68).

Nickel chloride (NiCl_2_) has been shown to substantially impair the immunoadhesive activity of chicken erythrocytes. Empirical evidence indicates that dietary supplementation with nickel chloride at concentrations of 300, 600, and 900 mg/kg NiCI2 feed results in a marked reduction in total erythrocyte count (TEC), hemoglobin (Hb) concentration, and packed cell volume (PCV) in broilers, while concomitantly increasing erythrocyte osmotic fragility (EOF), thereby indicating significant hematological alterations. Particularly in groups receiving 600 and 900 mg/kg, erythrocyte immunoadhesive function was notably compromised, as demonstrated by a significant decrease in erythrocyte C3b receptor rosette rate (E-C3bRR) and an increase in erythrocyte immune complex rosette rate (E-ICRR) (69). Additionally, diets high in fluoride have similarly been observed to decrease E-C3bRR and increase E-ICRR in broiler erythrocytes (70). In contrast, Astragalus polysaccharide (APS) has been found to significantly enhance E-C3bRR and E-ICRR in chickens infected with Infectious bursal disease virus (IBDV), thereby effectively restoring erythrocyte immunoadhesive function (9).

## 5 Erythrocytes in pathogen recognition and responses

**Figure 2:**
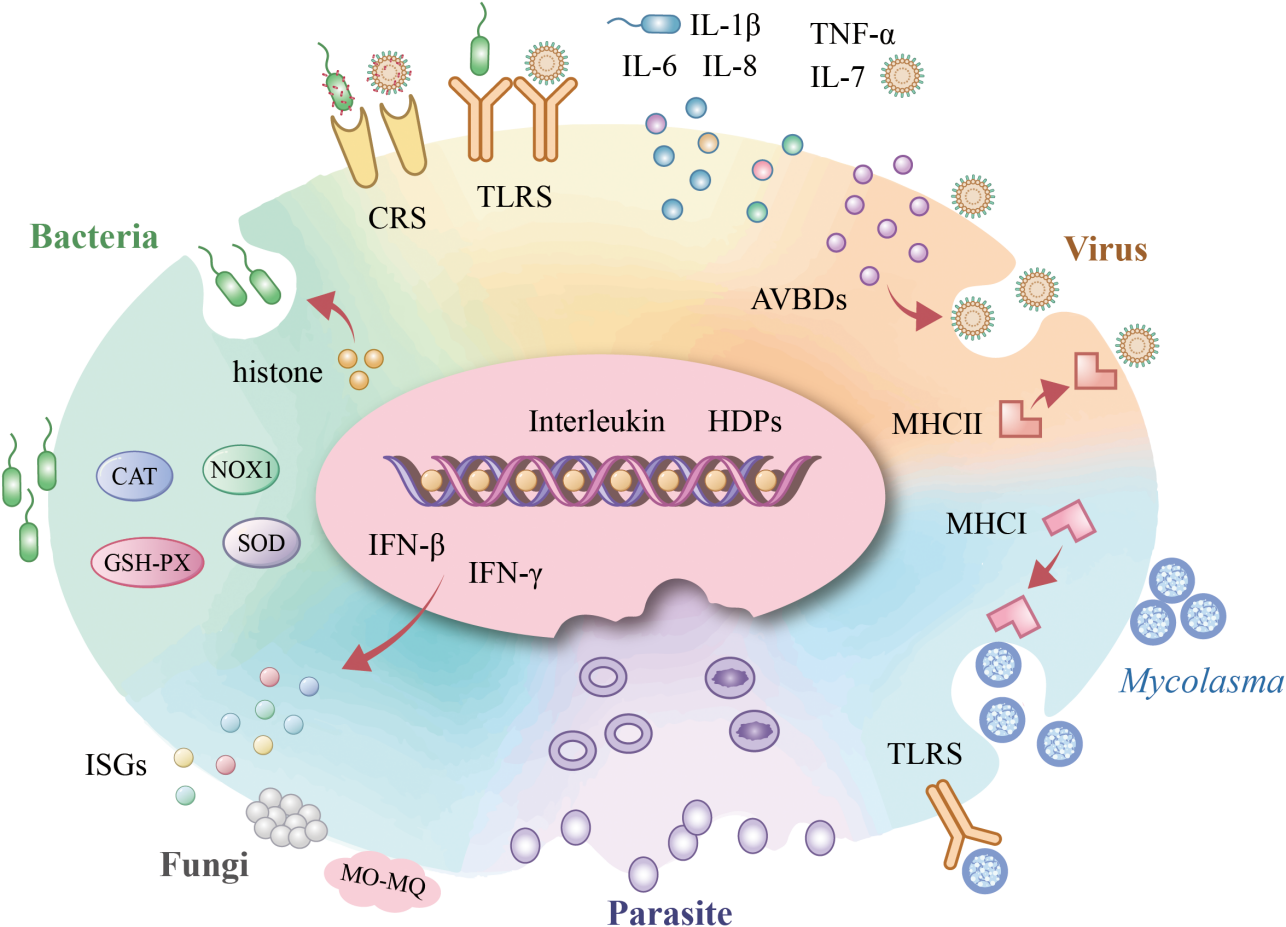
Avain erythrocyte involvement in pathogen infections. Upon bacterial infection, avian erythrocytes activate TLR-mediated pattern recognition, initiating IFN-β-dependent defense mechanisms that orchestrate interleukin responses. Concurrently, they exert histone-driven antimicrobial actions and enhance bacterial phagocytosis via the complement receptor system (CRs) while maintaining redox homeostasis through antioxidant enzymes. During viral challenge, TLR engagement similarly triggers cytokine modulation, with AVBD secretion providing direct antiviral activity alongside CRs-facilitated opsonization and MHC-II-restricted intracellular viral clearance. *Mycoplasma* elimination occurs through TLR-initiated signaling coupled with MHC-I-dependent pathogen eradication. For fungal pathogens (e.g., *Candida albicans*), erythrocytes deploy IFN-γ-mediated defenses that potentiate monocyte/macrophage (Mo-MØ) phagocytic activity. In malaria infection, erythrocytes establish an immune-privileged microenvironment that sequesters *Plasmodium parasites*.

Erythrocytes play a crucial role in pathogen recognition and immune responses. Recent studies have revealed that erythrocytes participate actively in defending against various pathogens, including viruses, bacteria, fungi, *Mycoplasma*, and parasites. These cells not only recognize pathogens through surface receptors but also initiate immune responses, modulate inflammation, and contribute to pathogen clearance. This section explores the multifaceted roles of erythrocytes in different infectious contexts, highlighting their dynamic involvement in host defense mechanisms (**Figure 2**).

### 5.1 Virus

Chicken erythrocytes are pivotal in the antiviral immune response, utilizing various mechanisms such as TLR signaling, cytokine production, antimicrobial peptide secretion, complement activation, and immune modulation. Upon viral infection, avian erythrocytes initiate TLR signaling pathways. In chickens infected with MDV, there is a significant upregulation in the expression of *TLR2*, *TLR3*, *TLR4*, *TLR6*, major histocompatibility *MHC II*, *IL-7*, *IFN-β*, chicken myelomonocytic growth factor (*cMGF*), and *MyD88* in erythrocytes. Conversely, the expression levels of *TLR5*, *TLR7*, *IL-12*, *IL-13*, and *IFN-α* are significantly downregulated in these cells. In contrast, the expression of *TLR1*, *TLR15*, *TLR21*, major histocompatibility *MHC I*, and tumor necrosis factor receptor-associated factor 6 (*TRAF6*) remains unchanged (8). In erythrocytes infected with the avian influenza virus subtype H9N2, the mRNA expression of NF-κB pathway components and other immune-related genes is significantly upregulated at 2 hours post-infection. However, at this time point, *TRAF6* expression is notably downregulated (3).

Avian erythrocytes play a pivotal role in modulating cytokine expression following viral infections. These cells contribute to the activation of crucial signal transduction pathways, notably the NF-κB pathway, in response to viral challenges. Such activation results in the expression of pro-inflammatory cytokines, including *TNF-α*, *IL-1β*, and *IL-6*, which are instrumental in promoting inflammation and are vital for the control of viral replication (3). Additionally, in the context of MDV infection, chicken erythrocytes are involved in the host immune response through the regulation of genes associated with type I interferons, defensins, and the prostagland in metabolic pathway. Notably, within these erythrocytes, there is a significant upregulation of *IL-7*, *IFN-β*, and chicken myelomonocytic growth factor (cMGF), alongside a marked downregulation of *IL-12* and *IL-13* expression (8). Conversely, erythrocytes from chickens in the early stages of Thiram-induced TD show a significant reduction in *IL-7* expression. Previous research has highlighted the critical role of IL-7 in cell survival and proliferation, mediated through the phosphatidylinositol-3-kinase (PI3K)/AKT signaling pathway (71). Consequently, the reduced *IL-7* expression observed in early TD may contribute to apoptosis in certain cell populations (33).

Chicken erythrocytes are integral to the host immune response triggered by MDV. Studies have demonstrated that these erythrocytes inherently express eight avian β-defensins (*AvBDs*) (specifically *AvBD1* through *AvBD7* and *AvBD9*) and liver-expressed antimicrobial peptide 2 (*LEAP-2*). Upon MDV infection, there is a marked upregulation in the expression levels of *AvBD2*, *AvBD4*, and *AvBD7*, while *AvBD1*, *AvBD6*, and *AvBD9* expression levels are significantly downregulated (10). Additionally, chicken erythrocytes produce antimicrobial peptides, such as AvBD11, in response to infections. These peptides are directly involved in antimicrobial defense, are upregulated following viral infection, and influence the host’s innate immune response. This influence is mediated through the promotion of cytokine production (including TNF, IFN-γ, IL-1β, and IL-10) and the facilitation of pathogen clearance (5). By synthesizing pro-inflammatory cytokines and antimicrobial peptides, and activating immune signaling pathways, erythrocytes substantially enhance the host’s capacity to combat viral infections and decrease viral load.

Erythrocytes engage complement receptors to enhance the clearance of pathogens. Empirical evidence indicates that the *in vitro* interaction of the H9N2 virus with chicken erythrocytes induces the expression of complement-related genes, including *C1s*, *C1q*, *C2*, *C3*, *C3AR1*, *C4*, *C4A*, *C5*, *C5AR1*, *C7*, *CD93*, and *CFD* within these cells. Notably, the expression levels of *C1q*, *C4*, *C1s*, *C2*, *C3*, *C5*, *C7*, and *CD93* are significantly upregulated at 2 hours post-interaction but are markedly downregulated at 10 hpi. Additionally, the expression levels of *C1q*, *C4*, *C1s*, *C2*, *C3*, *C5*, *C7*, *CFD*, *C3AR1*, *C4A*, (72) and *C5AR1* are significantly upregulated on day 7 post-interaction. Jiang et al. (9) reported that infection with IBDV significantly suppresses critical erythrocyte immune parameters, including C3b receptor expression, erythrocyte immune complex rosette formation rate, and phagocytic capacity. Treatment with an appropriate dose of APS effectively restores C3bR expression and enhances phagocytic activity.

### 5.2 Bacteria

Upon stimulation by various bacterial species, including *S.aureus*, *A. hydrophliai*, and *E. coli*, goose erythrocytes demonstrated differential upregulation of *TLR4*, *IL-1β*, *IL-6*, and *IL-8*. This suggests the involvement of goose erythrocytes in the immune response subsequent to bacterial infection (6). Bacterial stimulation led to a significant increase in the production of ROS and inducible nitric oxide synthase (iNOS) within goose erythrocytes. Moreover, the expression levels of *NOX1* and *NOX5*, members of the NADPH oxidase (NOX) family, were markedly upregulated, indicating their potential role in regulating ROS. In response to bacterial stress, the expression of antioxidant enzymes, including SOD, catalase (CAT), and glutathione peroxidase (GSH-Px), were elevated in goose erythrocytes, thereby enhancing the antioxidant defense system’s capacity to effectively scavenge ROS (73).

The histone complex, comprising H1, H2A, H2B, H3, H4, and H5, isolated from chicken erythrocytes, demonstrates antimicrobial efficacy against a range of Gram-negative and Gram-positive planktonic bacteria (74). An investigation into the antimicrobial biofilm activity of poultry erythrocyte histones against methicillin-sensitive *S.aureus* (MSSA) and methicillin-resistant *S.aureus* (MRSA) indicated that both histones and indolicidin (used as a positive control) upregulated the expression of apsS and apsR, genes involved in the antimicrobial peptide (AMP) sensor/regulatory system in *S.aureus*. Furthermore, the expression of dltB and vraF, genes associated with AMP resistance mechanisms, was induced by histones in biofilm-embedded bacterial cells. The bactericidal kinetics of histones against *S.aureus* revealed a rapid bactericidal effect, occurring in less than five minutes. Notably, the erythrocyte-specific histone H5, when purified, exhibited a 3–4 fold enhancement in antimicrobial activity against planktonic cells compared to the histone mixture. Moreover, research indicates that purified histone H5 derived from chicken erythrocytes exhibits broad-spectrum antimicrobial properties, notably against vancomycin-resistant *enterococci* (VRE), and effectively disrupts biofilm formation by *Listeria* and *Pseudomonas aeruginosa* (11). In addition to AMPs, hemoglobin, the predominant protein in erythrocytes, also serves as an antimicrobial agent. Previous studies have demonstrated that hemoglobin can elicit antimicrobial activity by generating reactive oxygen species during pathogenic assaults and plays a role in pathogen clearance from the bloodstream through the process of hemoglobin oxygenation (75).

Liu et al. (7) investigate the effects of avian pathogenic *E. coli* (APEC) on the expression of complement genes in chicken erythrocytes. *In vitro* studies demonstrated that *E. coli* adhered to erythrocytes, as confirmed by immunofluorescence and scanning electron microscopy. Real-time PCR analysis indicated an initial downregulation of *C4*, *C4A*, and *MBL*, followed by an upregulation of *CD93*, *C1q*, *C1s*, *C3*, *C3AR1*, *C5AR1*, and *C6* over time. *In vivo* experiments showed early activation of complement genes such as *C4* and *C4A*, with significant upregulation of classical pathway components (*C1q*, *C3*) by day 7. Conversely, the expression of MBL, a marker of the lectin pathway, was suppressed, suggesting inhibition of the lectin pathway and activation of the classical pathway during recovery. These findings provide novel insights into erythrocyte-pathogen interactions and the dynamics of the complement system in bacterial infections.

### 5.3 Fungi

Chicken erythrocytes have the capacity to form rosette structures with monocytes (Mo) and macrophages (MØ), leading to the activation of macrophages through the release of soluble molecules, thereby augmenting their phagocytic capacity. The phagocytic function of the Mo-MØ system has been evolutionarily conserved. Upon co-culturing chicken erythrocytes with *C.albicans*, it is possible that cytokine-like factors exhibiting interferon-gamma (IFN-γ)-like activity are released, which may demonstrate migratory inhibitory factor activity (12). IFN-γ is a highly pleiotropic cytokine known for its pro-inflammatory and antiviral properties, and it has been identified in the nucleated erythrocytes of mice, where it plays a role in regulating the development and function of monocyte/macrophage lineages (76, 13). Avian erythrocytes enhance the phagocytic activity of Mo-MØ against *C. albicans* (12).

### 5.4 Mycoplasma

Chicken erythrocytes play a critical immune role in *Mycoplasma* infection through the activation of the TLR signaling pathway. Lam (77) observed that following a 5-hour co-culture of chicken erythrocytes with *Mycoplasma gallisepticum* (*M. gallisepticum*), the erythrocytes exhibited a reduction in size and a loss of their typical ovoid morphology. The cell surfaces displayed extensive folding and indentations, with the presence of perforations or holes. These morphological alterations suggest that *M. gallisepticum* may adhere to and penetrate the erythrocytes, resulting in perforation and subsequent hemolysis. Furthermore, Helmy et al. (78) identified two novel and potent inhibitors of *M. gallisepticum* growth in poultry, highlighting their significant potential in controlling avian mycoplasmosis and contributing to the development of new antimicrobial agents.

Han et al. (22) reported analogous morphological alterations in erythrocytes subsequent to infection with *Mycoplasma synoviae* (*M. synoviae*). The study further identified particles resembling *M. synoviae* adhered to the erythrocyte surfaces, suggesting a direct interaction between the pathogen and the host cells. Notably, there was a significant upregulation in the mRNA expression of immune-related genes, including *TLR1*, *TLR2*, *TLR3*, *TLR4*, *TLR5*, *TLR7*, *TLR15*, *MHC I*, *MHC II*, and *MyD88* at various time intervals. This upregulation was particularly pronounced for *TLR1*, *TLR2*, *TLR3*, *TLR15*, and *MHC II* at 6 and 10 hours post-infection. These results provide the inaugural evidence of *M. synoviae*-induced changes in erythrocyte morphology and the activation of TLR signaling pathways, implying a role for erythrocytes in the early immune response to *M. synoviae* infection.

### 5.5 Parasite

Pendl et al. (79) elucidated a novel pathogenic mechanism of *Plasmodium matutinum* (*P. matutinum*) (pLINN1 lineage) in Turdus pilaris. Their study demonstrated that despite a low parasitemia level in erythrocytes (0.3%), the parasite employs several strategies to evade host immune clearance. Specifically, it completes schizont development within the mononuclear phagocyte system, thereby circumventing erythrocyte immune surveillance. Additionally, the parasite’s distinct vacuolar morphology, measuring up to 1.5 μm in diameter, likely impedes immune recognition. Furthermore, the development of elongated schizonts, reaching up to 47 μm within endothelial cells, leads to vascular blockage and the establishment of an immune-privileged microenvironment. These findings offer valuable insights into the immune evasion strategies employed by *Plasmodium* in non-adapted hosts and underscore the critical role of extracellular development in the disease’s pathogenesis.

Himmel et al. (80) conducted a study on natural *Plasmodium* infections in *Turdus merula* and *Turdus philomelos*, emphasizing the assessment of parasite load and its histopathological effects on host organs. The findings indicated that *P. matutinum* LINN1 is a prevalent parasite lineage characterized by a substantial extracellular erythrocytic parasite load, potentially triggering host immune evasion mechanisms. Birds infected with *P. matutinum* LINN1 demonstrated elevated parasite loads, frequently accompanied by histopathological alterations such as increased extracellular parasite numbers, inflammation, and tissue necrosis. Conversely, birds infected with other *Plasmodium* lineages exhibited lower parasite loads. Additionally, the study identified that *Leucocytozoon* sp. infection prompts the formation of giant cells, which are implicated in immune evasion by possibly suppressing the host’s immune response to prevent pathogen clearance. These results suggest that distinct parasite lineages may utilize varied immune evasion strategies, thereby influencing the host’s immune response and ultimately impacting the host’s health and survival.

## 6 Perspectives and conclusion

In conclusion, this study systematically elucidates the role of avian erythrocytes in innate immunity and inflammatory responses. Unlike their mammalian counterparts, avian erythrocytes, as the most abundant cells in the circulatory system, retain nuclei and functional organelles. This cellular architecture enables them to respond to pathogen-associated molecular patterns through transcription and translation, thereby conferring immunocompetence. The anti-infection mechanisms of avian erythrocytes encompass nucleus-dependent synthesis and secretion of immune molecules, efficient pathogen capture and targeted clearance, and precise regulation of inflammatory and immune homeostasis. While this review highlights various immunomodulatory functions of avian erythrocytes, the comprehensive scope of their involvement in avian immune responses remains to be fully elucidated.

Avian erythrocytes demonstrate functional immunological similarities with those of other species, including humans. Similar to nucleated erythrocytes in humans and piscine erythrocytes, avian erythrocytes express multiple TLRs (24, 25). However, unlike their mammalian counterparts, TLR9 expression has not yet been reported on avian erythrocytes. Instead, chTLR21 on erythrocytes activates an NF-κB signaling cascade analogous to that triggered by TLR9 in humans, although it operates through a unique TBK1 kinase-independent mechanism (44). Upon exogenous stimulation, avian erythrocytes initiate TLR-mediated signaling to express cytokines and engage in immune responses, a process also observed in fish (81). The involvement of avian erythrocytes in oxidative stress response is consistent with findings in human, murine, and fish erythrocytes (82, 83, 84). Additionally, complement receptors expressed on avian erythrocytes facilitate immune clearance, reflecting their function in human and murine systems (28, 85).

Future research will be crucial for understanding avian diseases like Marek's disease and tibial dyschondroplasia and for developing new strategies to reduce economic losses in the poultry industry from inflammation caused by avian bacterial or viral infections. Considering the continuously expanding repertoire of immune functions identified in avian and fish erythrocytes, a comparative evolutionary biology approach holds significant potential for uncovering deeper, yet unrecognized, immunological roles of erythrocytes in both avian and human physiology. However, systematic investigations into the immune functions of avian erythrocytes remain limited, resulting in substantial knowledge gaps. While research on human nucleated erythrocytes has identified specific subpopulations that mediate immune functions (86), it remains unclear which erythrocyte subpopulations in avian species, and at what stages of differentiation, modulate immune responses upon pathogen challenge. The comprehensive molecular profile of such immunomodulation has yet to be fully elucidated. While poly(I:C) stimulation has been demonstrated to modify chromatin architecture in erythrocytes (87), the mechanisms underlying chromatin remodeling in response to various pathogenic infections and the impact of metabolic reprogramming on immune function remain largely unexplored. In the realm of human erythrocyte immunology, proteomic and metabolomic methodologies have yielded further insights into the immune functions of erythrocytes (88). Nonetheless, the application of these advanced technologies to avian erythrocytes is limited. Previous transcriptomic analyses in avian models have revealed immune regulatory mechanisms in chicken erythrocytes (21, 89, 90). Future research should adopt an integrated approach that utilizes cutting-edge biotechnological tools, such as single-cell transcriptomics and proteomics, to systematically examine the immune responses of avian erythrocytes during infection.
